# Could it be a secondary hypertension?

**DOI:** 10.1186/1824-7288-41-S2-A49

**Published:** 2015-09-30

**Authors:** Maria Chiara Matteucci

**Affiliations:** 1 Department of Nephrology and Urology, Bambino Gesù Children Research Hospital, Rome 00153, Italy

## 

High percentage of hypertensive children younger than 15 years of age show secondary hypertension. Correction of the underlying disorder may cure the hypertension and avoid the need for prolonged drug therapy.

When to suspect a secondary hypertension at initial evaluation:

• No family history of hypertension in a thin child

• Age younger than 10 years and prepubertal stage

• Acute rise in blood pressure previously normal

• Severe hypertension (SBP/DBP ≥ 99th percentile plus 5 mmHg)

• Mild hypertension (SBP/DBP ≥ 95th percentile plus 5 mmHg) in systemic disease

• Nocturnal hypertension, reduced nocturnal dipping or diastolic hypertension at 24-hour BP monitoring

• Urological or congenital renal pathology

• Symptons of sympathetic overactivity

• Ambigous genitalia

• Edema, high serum creatinine and/or abnormal urinalysis

• Systemic disorders with secondary glomerulonephritis

• Neonatal umbelical catheterization

• Abdominal auscultatory bruit

• Upper extremities hypertension and lower extremities hypotension.

• Familiar chronic or congenital renal disease

If suspicion is consistent for a secondary hypertension the following diagnostic studies are needed:

• Renal ultrasonography as recommended by the National High Blood Pressure Education Program Working Group in Children and adolescent (NHBPEP), 2004 [1]

• 99mTc-dimercaptosuccinic acid (DMSA) renal scan, sensitive study for renal cortical loss and scarring

• Plasma renin activity

• Plasma and urine catecholamines

• Renovascular imaging as magnetic resonance angiography, computed tomographic angiography, duplex Doppler ultrasonography and standard intraarterial angiography

• Echocardiography
